# 4-(4-Bromo­benzyl­ideneamino)-3-{1-[4-(2-methyl­prop­yl)phen­yl]eth­yl}-1-(mor­phol­ino­meth­yl)-1*H*-1,2,4-triazole-5(4*H*)-thione

**DOI:** 10.1107/S160053680802254X

**Published:** 2008-07-23

**Authors:** Hoong-Kun Fun, Samuel Robinson Jebas, P. S. Patil, B. Kalluraya, A. Muralidharan

**Affiliations:** a X-ray Crystallography Unit, School of Physics, Universiti Sains Malaysia, 11800 USM, Penang, Malaysia; b Department of Studies in Physics, Mangalore University, Mangalagangotri, Mangalore 574 199, India; c Department of Studies in Chemistry, Mangalore University, Mangalagangotri, Mangalore 574 199, India; d Department of Chemistry, Nehru Arts and Science College, Kanhangad, Kerala 671 328, India

## Abstract

There are two mol­ecules (*A* and *B*) in the asymmetric unit of the title compound, C_26_H_32_BrN_5_OS, with almost identical geometry. The morpholine ring adopts the usual chair conformation in both mol­ecules. The triazole ring forms dihedral angles of 4.84 (6) and 74.19 (6)°, respectively, with the bromo­phenyl and isobutylbenzene rings in mol­ecule *A*, and angles of 16.68 (7) and 87.29 (6)°, respectively, in mol­ecule *B*. Intra­molecular C—H⋯S hydrogen bonds generate *S*(5) and *S*(6) ring motifs in both independent mol­ecules. The crystal structure is stabilized by C—H⋯N, C—H⋯Br and C—H⋯O hydrogen-bonding inter­actions, together with C—H⋯π inter­actions.

## Related literature

For general background, see: Raman *et al.* (2004 ▶); Tramontini *et al.* (1988 ▶); Tramontini & Angliolini (1990 ▶); Lopes *et al.* (2004 ▶); Joshi *et al.* (2004 ▶); Ferlin *et al.* (2002 ▶); Holla *et al.* (2003 ▶); Malinka *et al.* (2005 ▶); Karthikeyan *et al.* (2006 ▶); Palaska *et al.* (2002 ▶). For related structures, see: Fun, Jebas, Razak *et al.* (2008 ▶); Fun, Jebas, Sujith *et al.* (2008 ▶). For bond-length data, see: Allen *et al.* (1987 ▶). For ring puckering analysis, see: Cremer & Pople (1975 ▶). For graph-set analysis of hydrogen bonding, see: Bernstein *et al.* (1995 ▶).
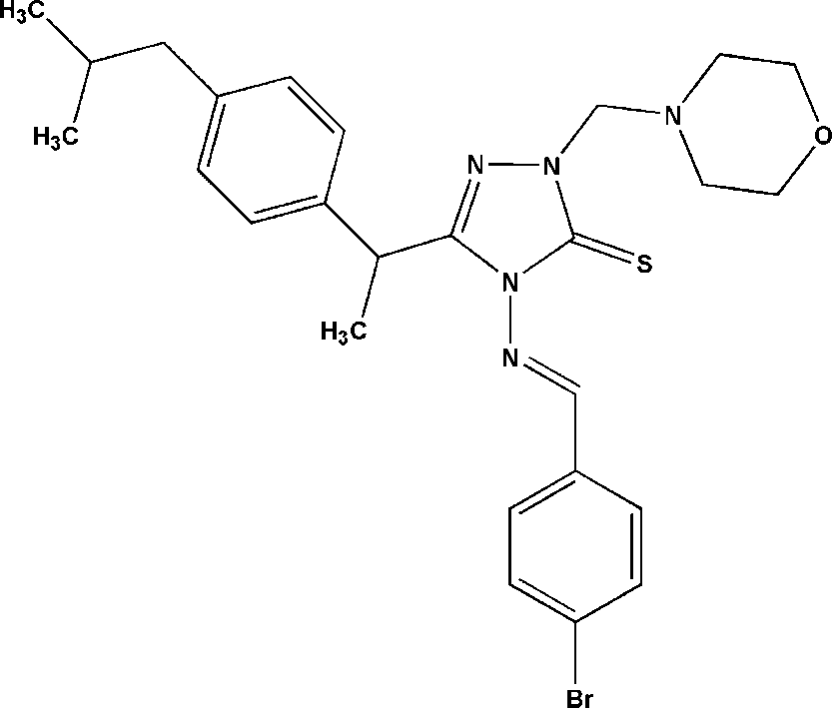


## Experimental

### 

#### Crystal data

C_26_H_32_BrN_5_OS
*M* *_r_* = 542.54
Triclinic, 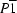

*a* = 10.1381 (1) Å
*b* = 17.0356 (2) Å
*c* = 17.2077 (2) Å
α = 64.168 (1)°
β = 79.773 (1)°
γ = 78.816 (1)°
*V* = 2609.55 (6) Å^3^
*Z* = 4
Mo *K*α radiation
μ = 1.68 mm^−1^
*T* = 100.0 (1) K
0.45 × 0.34 × 0.26 mm

#### Data collection

Bruker SMART APEXII CCD area-detector diffractometer
Absorption correction: multi-scan (*SADABS*; Bruker, 2005 ▶) *T*_min_ = 0.522, *T*_max_ = 0.639
89536 measured reflections
18804 independent reflections
13217 reflections with *I* > 2σ(*I*)
*R*_int_ = 0.044

#### Refinement

*R*[*F* ^2^ > 2σ(*F* ^2^)] = 0.039
*wR*(*F* ^2^) = 0.097
*S* = 1.02
18804 reflections
619 parameters
H-atom parameters constrained
Δρ_max_ = 1.33 e Å^−3^
Δρ_min_ = −0.84 e Å^−3^

### 

Data collection: *APEX2* (Bruker, 2005 ▶); cell refinement: *APEX2*; data reduction: *SAINT* (Bruker, 2005 ▶); program(s) used to solve structure: *SHELXTL* (Sheldrick, 2008 ▶); program(s) used to refine structure: *SHELXTL*; molecular graphics: *SHELXTL*; software used to prepare material for publication: *SHELXTL* and *PLATON* (Spek, 2003 ▶).

## Supplementary Material

Crystal structure: contains datablocks global, I. DOI: 10.1107/S160053680802254X/ci2634sup1.cif

Structure factors: contains datablocks I. DOI: 10.1107/S160053680802254X/ci2634Isup2.hkl

Additional supplementary materials:  crystallographic information; 3D view; checkCIF report

## Figures and Tables

**Table 1:** Hydrogen-bond geometry (Å, °)

*D*—H⋯*A*	*D*—H	H⋯*A*	*D*⋯*A*	*D*—H⋯*A*
C7*A*—H7*AA*⋯S1*A*	0.93	2.46	3.195 (2)	137
C9*A*—H9*AB*⋯S1*A*	0.97	2.86	3.252 (2)	105
C4*B*—H4*BA*⋯N5*A*^i^	0.93	2.56	3.384 (2)	147
C10*A*—H10*A*⋯Br1*A*^ii^	0.97	2.86	3.770 (2)	158
C7*B*—H7*BA*⋯S1*B*	0.93	2.56	3.190 (2)	125
C9*B*—H9*BB*⋯S1*B*	0.97	2.85	3.254 (2)	106
C15*A*—H15*A*⋯O1*A*^iii^	0.98	2.29	3.244 (2)	165
C4*A*—H4*AA*⋯*Cg*1^ii^	0.93	2.53	3.401 (2)	156

Symmetry codes: (i) 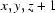; (ii) 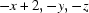; (iii) 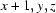. *Cg*1 is the centroid of the C16*A*–C21*A* ring.

**Fig. 1.**
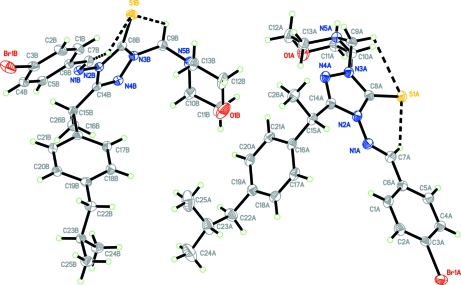
The asymmetric unit of the title compound. Displacement ellipsoids are drawn at the 50% probability level. Hydrogen bonds are shown as dashed lines.

**Fig. 2.**
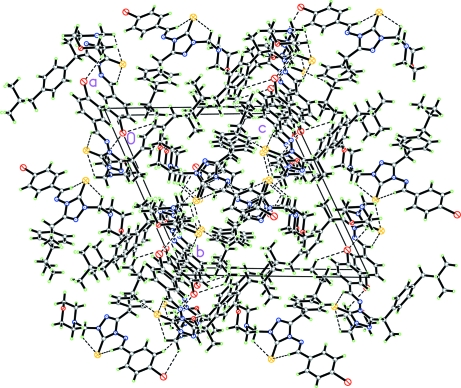
The crystal packing of the title compound, viewed along the a axis. Hydrogen bonds are shown as dashed lines.

## supplementary crystallographic information

### Comment

Mannich reaction is a three-component condensation reaction involving active hydrogen containing compound, formaldehyde and a secondary amine (Raman *et al.*, 2004). The amino alkylation of aromatic substrates by Mannich reaction is of considerable importance for the synthesis and modification of biologically active compounds (Tramontini & Angliolini, 1990). Mannich bases have been reported as potential biological agents. They find applications as antitubercular (Joshi *et al.*, 2004), antimalarial (Lopes *et al.*, 2004), vasorelaxing (Ferlin *et al.*, 2002), anticancer (Holla *et al.*, 2003), and analgesic drugs (Malinka *et al.*, 2005). They are also used in polymer industry as paints and surface active agents (Tramontini *et al.*, 1988). Some Mannich bases are reported to exhibit activity *in vitro* against murine P388 lymphocytic leukemia cells (Karthikeyan *et al.*, 2006). Similarly, ibuprofen belongs to the class of Non-Steroidal Anti-Inflammatory Drugs (NSAIDs) with antipyretic, anti-inflammatory and analgesic properties (Palaska *et al.*, 2002). Previously, we have reported crystal structures of triazole derivatives containing a ibuprofen moiety (Fun, Jebas, Razak *et al.*, 2008; Fun, Jebas, Sujith *et al.*, 2008). In continuation of our work, we report here the crystal structure of the title compound.

The asymmetric unit of the title compound contains two crystallographically independent molecules (A and B) with almost similar geometries. The bond lengths and angles are found to have normal values (Allen *et al.*, 1987). The triazole rings in both molecules are planar with maximum deviations of 0.013 (2) Å and 0.023 (2) Å, respectively, for atoms C8A and N2B. The morpholine ring in both molecules adopt the usual chair conformation with puckering parameters Q = 0.578 (2) Å, θ = 176.6 (2)° and φ=152 (3)° in molecule A, and Q = 0.571 (2) Å,θ = 2.0 (2)° and φ = 70 (5)° in molecule B (Cremer & Pople, 1975). The N2A/C8A/N3A/N4A/C14A plane forms dihedral angles of 4.84 (6)° and 74.19 (6)°, respectively, with the C1A–C6A and C16A–C21A plane. The dihedral angle formed by the N2B/C8B/N3B/N4B/C14B plane with the C1B–C6B and C16B–C21B planes are 16.68 (7)° and 87.29 (6)°, respectively. Intramolecular C—H···S hydrogen bonds generate S(5) and S(6) ring motifs in both molecules (Bernstein *et al.*, 1995).

The crystal packing is stabilized by intermolecular C—H···N, C—H···Br and C—H···O hydrogen bonding interactions together with C—H···π interactions.

### Experimental

The title Mannich-base compound was obtained by the aminomethylation of its corresponding Schiff base, which was in turn obtained by refluxing a mixture of 4-amino-5-[1-(4-isobutylphenyl)ethyl]-4*H*-1,2,4-triazole-3-thiol (0.01 mol), 4-bromo-benzaldehyde (0.01 mol) in ethanol (50 ml) and 3 drops of concentrated H_2_SO_4_ for 3 h. A mixture of the above Schiff base (0.01 mol), formaldehyde (40%, 1 ml) and morpholine (0.01 mol) in ethanol (50 ml) was stirred at room temperature for 20 h. The solid product obtained was collected by filtration, washed with ethanol and dried. It was then recrystallized using ethanol. Single crystals suitable for X-ray analysis were obtained from acetone-*N*,*N*-dimethylformamide (DMF) (1:3) solution by slow evaporation (yield 85%; m.p. 383 K). Analysis for C_26_H_32_N_5_BrOS found (calculated%): C 57.55 (57.564), H 5.81 (5.904), N 12.82 (12.915), S 5.82 (5.904).

### Refinement

H atoms were positioned geometrically (C-H = 0.93-0.98 Å) and refined using a riding model, with *U*_iso_(H) = 1.2-1.5 *U*_eq_(C). A rotating group model was used for the methyl groups.

### Crystal data

**Table:**

C_26_H_32_BrN_5_OS	*Z* = 4
*M**_r_* = 542.54	*F*_000_ = 1128
Triclinic, *P*1	*D*_x_ = 1.381 Mg m^−^^3^
Hall symbol: -P 1	Mo *K*α radiation λ = 0.71073 Å
*a* = 10.1381 (1) Å	Cell parameters from 9909 reflections
*b* = 17.0356 (2) Å	θ = 2.2–29.6º
*c* = 17.2077 (2) Å	µ = 1.68 mm^−^^1^
α = 64.168 (1)º	*T* = 100.0 (1) K
β = 79.773 (1)º	Block, colourless
γ = 78.816 (1)º	0.45 × 0.34 × 0.26 mm
*V* = 2609.55 (6) Å^3^	

### Data collection

**Table:**

Bruker SMART APEXII CCD area-detector diffractometer	18804 independent reflections
Radiation source: fine-focus sealed tube	13217 reflections with *I* > 2σ(*I*)
Monochromator: graphite	*R*_int_ = 0.044
*T* = 100.0(1) K	θ_max_ = 32.5º
φ and ω scans	θ_min_ = 1.3º
Absorption correction: multi-scan(SADABS; Bruker, 2005)	*h* = −15→14
*T*_min_ = 0.523, *T*_max_ = 0.639	*k* = −25→25
89536 measured reflections	*l* = −26→25

### Refinement

**Table:**

Refinement on *F*^2^	Secondary atom site location: difference Fourier map
Least-squares matrix: full	Hydrogen site location: inferred from neighbouring sites
*R*[*F*^2^ > 2σ(*F*^2^)] = 0.039	H-atom parameters constrained
*wR*(*F*^2^) = 0.097	*w* = 1/[σ^2^(*F*_o_^2^) + (0.038*P*)^2^ + 1.3091*P*] where *P* = (*F*_o_^2^ + 2*F*_c_^2^)/3
*S* = 1.02	(Δ/σ)_max_ = 0.001
18804 reflections	Δρ_max_ = 1.33 e Å^−^^3^
619 parameters	Δρ_min_ = −0.84 e Å^−^^3^
Primary atom site location: structure-invariant direct methods	Extinction correction: none

### Special details

**Table:**

Experimental. The data was collected with the Oxford Cyrosystem Cobra low-temperature attachment.
Geometry. All e.s.d.'s (except the e.s.d. in the dihedral angle between two l.s. planes) are estimated using the full covariance matrix. The cell e.s.d.'s are taken into account individually in the estimation of e.s.d.'s in distances, angles and torsion angles; correlations between e.s.d.'s in cell parameters are only used when they are defined by crystal symmetry. An approximate (isotropic) treatment of cell e.s.d.'s is used for estimating e.s.d.'s involving l.s. planes.
Refinement. Refinement of *F*^2^ against ALL reflections. The weighted *R*-factor *wR* and goodness of fit *S* are based on *F*^2^, conventional *R*-factors *R* are based on *F*, with *F* set to zero for negative *F*^2^. The threshold expression of *F*^2^ > σ(*F*^2^) is used only for calculating *R*-factors(gt) *etc*. and is not relevant to the choice of reflections for refinement. *R*-factors based on *F*^2^ are statistically about twice as large as those based on *F*, and *R*- factors based on ALL data will be even larger.

### Fractional atomic coordinates and isotropic or equivalent isotropic displacement parameters (Å^2^)

**Table:**

	*x*	*y*	*z*	*U*_iso_*/*U*_eq_	
Br1A	1.463972 (19)	−0.136890 (12)	−0.024526 (12)	0.02730 (5)	
S1A	0.72929 (5)	0.25031 (3)	−0.21857 (3)	0.02396 (9)	
O1A	0.19942 (14)	0.30933 (10)	−0.05409 (9)	0.0338 (3)	
N1A	0.94677 (15)	0.20300 (9)	−0.06764 (9)	0.0216 (3)	
N2A	0.83105 (14)	0.26411 (9)	−0.08571 (9)	0.0195 (3)	
N3A	0.65694 (15)	0.35672 (9)	−0.13441 (9)	0.0201 (3)	
N4A	0.69613 (15)	0.37728 (9)	−0.07311 (9)	0.0217 (3)	
N5A	0.41603 (15)	0.40103 (9)	−0.15946 (9)	0.0220 (3)	
C1A	1.18024 (19)	0.07350 (12)	−0.02429 (11)	0.0256 (4)	
H1AA	1.1651	0.1122	0.0023	0.031*	
C2A	1.29078 (19)	0.00937 (13)	−0.00761 (11)	0.0266 (4)	
H2AA	1.3498	0.0041	0.0306	0.032*	
C3A	1.31323 (18)	−0.04743 (11)	−0.04847 (11)	0.0216 (3)	
C4A	1.22709 (19)	−0.04131 (11)	−0.10530 (11)	0.0231 (3)	
H4AA	1.2435	−0.0796	−0.1324	0.028*	
C5A	1.11568 (18)	0.02303 (11)	−0.12113 (11)	0.0235 (3)	
H5AA	1.0567	0.0277	−0.1591	0.028*	
C6A	1.09067 (18)	0.08075 (11)	−0.08100 (11)	0.0210 (3)	
C7A	0.96930 (18)	0.14595 (11)	−0.09930 (11)	0.0231 (3)	
H7AA	0.9088	0.1457	−0.1340	0.028*	
C8A	0.73866 (17)	0.28902 (11)	−0.14594 (10)	0.0196 (3)	
C9A	0.55336 (18)	0.41834 (11)	−0.18951 (11)	0.0231 (3)	
H9AA	0.5592	0.4770	−0.1958	0.028*	
H9AB	0.5749	0.4184	−0.2468	0.028*	
C10A	0.38410 (18)	0.32459 (11)	−0.16766 (11)	0.0229 (3)	
H10A	0.4380	0.2717	−0.1314	0.028*	
H10B	0.4062	0.3317	−0.2274	0.028*	
C11A	0.23609 (19)	0.31572 (13)	−0.14053 (12)	0.0281 (4)	
H11A	0.1826	0.3664	−0.1801	0.034*	
H11B	0.2168	0.2636	−0.1436	0.034*	
C12A	0.2238 (2)	0.38716 (15)	−0.05031 (14)	0.0332 (4)	
H12A	0.1942	0.3845	0.0075	0.040*	
H12B	0.1723	0.4380	−0.0911	0.040*	
C13A	0.37267 (19)	0.39652 (13)	−0.07209 (12)	0.0268 (4)	
H13A	0.3886	0.4495	−0.0693	0.032*	
H13B	0.4242	0.3466	−0.0304	0.032*	
C14A	0.80121 (18)	0.32077 (11)	−0.04479 (11)	0.0207 (3)	
C15A	0.88283 (18)	0.31567 (11)	0.02201 (11)	0.0221 (3)	
H15A	0.9783	0.3022	0.0034	0.027*	
C16A	0.84828 (17)	0.24255 (11)	0.11050 (11)	0.0210 (3)	
C17A	0.94838 (18)	0.17541 (13)	0.15147 (12)	0.0248 (4)	
H17A	1.0356	0.1739	0.1235	0.030*	
C18A	0.91887 (19)	0.11064 (13)	0.23385 (12)	0.0263 (4)	
H18A	0.9871	0.0663	0.2602	0.032*	
C19A	0.79030 (19)	0.11052 (12)	0.27771 (11)	0.0240 (3)	
C20A	0.68960 (18)	0.17745 (12)	0.23622 (11)	0.0235 (3)	
H20A	0.6024	0.1788	0.2642	0.028*	
C21A	0.71812 (18)	0.24208 (12)	0.15355 (12)	0.0231 (3)	
H21A	0.6494	0.2856	0.1266	0.028*	
C22A	0.7604 (2)	0.04019 (13)	0.36753 (12)	0.0316 (4)	
H22A	0.7927	−0.0169	0.3667	0.038*	
H22B	0.6632	0.0437	0.3823	0.038*	
C23A	0.8239 (2)	0.04639 (17)	0.43828 (13)	0.0390 (5)	
H23A	0.9221	0.0418	0.4230	0.047*	
C24A	0.7948 (3)	−0.0314 (2)	0.52512 (14)	0.0553 (7)	
H24A	0.8314	−0.0258	0.5699	0.083*	
H24B	0.8358	−0.0854	0.5209	0.083*	
H24C	0.6989	−0.0319	0.5389	0.083*	
C25A	0.7781 (3)	0.13307 (19)	0.44434 (17)	0.0565 (7)	
H25A	0.8002	0.1801	0.3893	0.085*	
H25B	0.8226	0.1353	0.4880	0.085*	
H25C	0.6820	0.1392	0.4596	0.085*	
C26A	0.8647 (2)	0.40454 (12)	0.02743 (13)	0.0291 (4)	
H26A	0.8920	0.4488	−0.0283	0.044*	
H26B	0.9192	0.4006	0.0696	0.044*	
H26C	0.7714	0.4200	0.0444	0.044*	
Br1B	0.48478 (3)	0.645886 (13)	0.647264 (13)	0.03762 (6)	
S1B	0.08036 (5)	0.55979 (3)	0.28269 (3)	0.02256 (9)	
O1B	0.35396 (15)	0.25009 (11)	0.22342 (11)	0.0440 (4)	
N1B	0.13059 (14)	0.44610 (9)	0.49490 (9)	0.0176 (3)	
N2B	0.07466 (14)	0.41862 (9)	0.44400 (8)	0.0166 (3)	
N3B	0.00212 (14)	0.39715 (9)	0.34664 (8)	0.0180 (3)	
N4B	−0.01975 (14)	0.32382 (9)	0.42336 (9)	0.0190 (3)	
N5B	0.09910 (15)	0.35433 (10)	0.22751 (9)	0.0219 (3)	
C1B	0.36569 (18)	0.59648 (11)	0.45716 (11)	0.0233 (3)	
H1BA	0.3785	0.6186	0.3969	0.028*	
C2B	0.42654 (19)	0.63059 (11)	0.49939 (11)	0.0256 (4)	
H2BA	0.4802	0.6753	0.4681	0.031*	
C3B	0.40603 (18)	0.59695 (11)	0.58892 (11)	0.0227 (3)	
C4B	0.32932 (17)	0.52864 (11)	0.63734 (11)	0.0212 (3)	
H4BA	0.3185	0.5060	0.6976	0.025*	
C5B	0.26936 (17)	0.49485 (11)	0.59445 (10)	0.0192 (3)	
H5BA	0.2181	0.4489	0.6261	0.023*	
C6B	0.28537 (17)	0.52939 (11)	0.50386 (10)	0.0183 (3)	
C7B	0.22076 (17)	0.49746 (11)	0.45565 (11)	0.0200 (3)	
H7BA	0.2453	0.5145	0.3964	0.024*	
C8B	0.05586 (16)	0.45842 (11)	0.35662 (10)	0.0178 (3)	
C9B	−0.01500 (17)	0.39801 (11)	0.26330 (10)	0.0206 (3)	
H9BA	−0.0941	0.3701	0.2710	0.025*	
H9BB	−0.0323	0.4587	0.2217	0.025*	
C10B	0.1158 (2)	0.25835 (12)	0.27558 (13)	0.0304 (4)	
H10C	0.0330	0.2359	0.2791	0.036*	
H10D	0.1354	0.2424	0.3342	0.036*	
C11B	0.2306 (2)	0.21881 (15)	0.22926 (16)	0.0406 (5)	
H11C	0.2414	0.1552	0.2603	0.049*	
H11D	0.2092	0.2337	0.1713	0.049*	
C12B	0.3396 (2)	0.34296 (16)	0.17616 (14)	0.0368 (5)	
H12C	0.3220	0.3580	0.1174	0.044*	
H12D	0.4235	0.3641	0.1729	0.044*	
C13B	0.22578 (18)	0.38805 (13)	0.21804 (12)	0.0258 (4)	
H13C	0.2472	0.3780	0.2747	0.031*	
H13D	0.2157	0.4510	0.1826	0.031*	
C14B	0.02267 (16)	0.33944 (10)	0.48161 (10)	0.0172 (3)	
C15B	0.01969 (17)	0.28116 (10)	0.57663 (10)	0.0182 (3)	
H15B	−0.0241	0.3166	0.6085	0.022*	
C16B	0.16129 (17)	0.24513 (10)	0.60310 (10)	0.0175 (3)	
C17B	0.26067 (18)	0.21508 (11)	0.55173 (11)	0.0213 (3)	
H17B	0.2406	0.2185	0.4996	0.026*	
C18B	0.38945 (18)	0.18011 (11)	0.57731 (11)	0.0224 (3)	
H18B	0.4543	0.1604	0.5419	0.027*	
C19B	0.42331 (17)	0.17395 (10)	0.65474 (10)	0.0198 (3)	
C20B	0.32294 (19)	0.20293 (11)	0.70662 (11)	0.0234 (3)	
H20B	0.3426	0.1986	0.7592	0.028*	
C21B	0.19414 (19)	0.23818 (11)	0.68119 (11)	0.0231 (3)	
H21B	0.1290	0.2574	0.7168	0.028*	
C22B	0.56389 (17)	0.13790 (11)	0.68160 (11)	0.0220 (3)	
H22C	0.5943	0.1779	0.6990	0.026*	
H22D	0.6242	0.1359	0.6318	0.026*	
C23B	0.57477 (17)	0.04546 (11)	0.75647 (11)	0.0195 (3)	
H23B	0.5192	0.0490	0.8079	0.023*	
C24B	0.52154 (19)	−0.01900 (11)	0.73412 (12)	0.0254 (4)	
H24D	0.5318	−0.0765	0.7808	0.038*	
H24E	0.5716	−0.0208	0.6819	0.038*	
H24F	0.4276	−0.0003	0.7255	0.038*	
C25B	0.72130 (19)	0.01507 (12)	0.77739 (12)	0.0267 (4)	
H25D	0.7277	−0.0423	0.8246	0.040*	
H25E	0.7505	0.0558	0.7934	0.040*	
H25F	0.7778	0.0126	0.7272	0.040*	
C26B	−0.06443 (18)	0.20662 (11)	0.60044 (11)	0.0236 (3)	
H26D	−0.1538	0.2312	0.5835	0.035*	
H26E	−0.0698	0.1717	0.6620	0.035*	
H26F	−0.0224	0.1703	0.5708	0.035*	

### Atomic displacement parameters (Å^2^)

**Table:**

	*U*^11^	*U*^22^	*U*^33^	*U*^12^	*U*^13^	*U*^23^
Br1A	0.02633 (10)	0.02249 (9)	0.02637 (9)	0.00006 (7)	−0.00519 (7)	−0.00446 (7)
S1A	0.0275 (2)	0.0264 (2)	0.0216 (2)	−0.00191 (17)	−0.00418 (16)	−0.01344 (17)
O1A	0.0275 (7)	0.0451 (8)	0.0330 (7)	−0.0110 (6)	0.0043 (6)	−0.0203 (6)
N1A	0.0193 (7)	0.0218 (7)	0.0243 (7)	−0.0027 (5)	−0.0016 (5)	−0.0103 (6)
N2A	0.0193 (7)	0.0206 (7)	0.0214 (7)	−0.0046 (5)	−0.0002 (5)	−0.0113 (5)
N3A	0.0212 (7)	0.0204 (7)	0.0199 (6)	−0.0037 (5)	−0.0009 (5)	−0.0095 (5)
N4A	0.0232 (7)	0.0220 (7)	0.0227 (7)	−0.0058 (6)	0.0005 (5)	−0.0118 (6)
N5A	0.0237 (8)	0.0210 (7)	0.0206 (7)	−0.0010 (6)	−0.0028 (5)	−0.0085 (6)
C1A	0.0271 (9)	0.0290 (9)	0.0259 (9)	−0.0028 (7)	−0.0024 (7)	−0.0167 (7)
C2A	0.0256 (9)	0.0331 (10)	0.0229 (8)	−0.0054 (7)	−0.0045 (7)	−0.0119 (7)
C3A	0.0225 (8)	0.0184 (8)	0.0200 (8)	−0.0031 (6)	−0.0016 (6)	−0.0043 (6)
C4A	0.0269 (9)	0.0192 (8)	0.0253 (8)	−0.0028 (7)	−0.0042 (7)	−0.0106 (7)
C5A	0.0246 (9)	0.0239 (8)	0.0257 (8)	−0.0029 (7)	−0.0062 (7)	−0.0125 (7)
C6A	0.0217 (8)	0.0195 (8)	0.0213 (8)	−0.0047 (6)	−0.0010 (6)	−0.0078 (6)
C7A	0.0227 (9)	0.0235 (8)	0.0257 (8)	−0.0050 (7)	−0.0029 (6)	−0.0118 (7)
C8A	0.0211 (8)	0.0194 (8)	0.0165 (7)	−0.0063 (6)	0.0006 (6)	−0.0054 (6)
C9A	0.0257 (9)	0.0179 (8)	0.0215 (8)	−0.0029 (7)	−0.0022 (6)	−0.0046 (6)
C10A	0.0240 (9)	0.0213 (8)	0.0219 (8)	−0.0004 (7)	−0.0042 (6)	−0.0078 (7)
C11A	0.0242 (9)	0.0336 (10)	0.0290 (9)	−0.0027 (7)	−0.0035 (7)	−0.0155 (8)
C12A	0.0237 (10)	0.0465 (12)	0.0350 (10)	0.0008 (8)	−0.0013 (8)	−0.0248 (9)
C13A	0.0241 (9)	0.0321 (10)	0.0273 (9)	0.0002 (7)	−0.0031 (7)	−0.0167 (8)
C14A	0.0224 (8)	0.0197 (8)	0.0222 (8)	−0.0066 (6)	0.0019 (6)	−0.0109 (6)
C15A	0.0184 (8)	0.0259 (9)	0.0276 (8)	−0.0038 (6)	−0.0013 (6)	−0.0162 (7)
C16A	0.0192 (8)	0.0252 (8)	0.0257 (8)	−0.0033 (6)	−0.0025 (6)	−0.0168 (7)
C17A	0.0154 (8)	0.0361 (10)	0.0315 (9)	0.0002 (7)	−0.0041 (7)	−0.0228 (8)
C18A	0.0234 (9)	0.0312 (10)	0.0300 (9)	0.0048 (7)	−0.0114 (7)	−0.0181 (8)
C19A	0.0266 (9)	0.0266 (9)	0.0248 (8)	−0.0023 (7)	−0.0067 (7)	−0.0153 (7)
C20A	0.0187 (8)	0.0290 (9)	0.0284 (9)	−0.0037 (7)	−0.0003 (6)	−0.0174 (7)
C21A	0.0189 (8)	0.0238 (8)	0.0293 (9)	0.0015 (6)	−0.0042 (6)	−0.0147 (7)
C22A	0.0373 (11)	0.0333 (10)	0.0267 (9)	−0.0062 (8)	−0.0083 (8)	−0.0123 (8)
C23A	0.0300 (11)	0.0637 (15)	0.0280 (10)	−0.0085 (10)	−0.0037 (8)	−0.0222 (10)
C24A	0.0545 (16)	0.080 (2)	0.0273 (11)	−0.0111 (14)	−0.0072 (10)	−0.0161 (12)
C25A	0.0701 (19)	0.0764 (19)	0.0456 (14)	−0.0289 (15)	0.0060 (12)	−0.0427 (14)
C26A	0.0287 (10)	0.0284 (9)	0.0376 (10)	−0.0061 (8)	−0.0023 (8)	−0.0198 (8)
Br1B	0.06026 (15)	0.02600 (10)	0.03119 (10)	−0.01396 (9)	−0.02188 (9)	−0.00635 (8)
S1B	0.0235 (2)	0.01844 (19)	0.02141 (19)	−0.00316 (16)	−0.00178 (15)	−0.00441 (15)
O1B	0.0279 (8)	0.0540 (10)	0.0624 (10)	0.0112 (7)	−0.0086 (7)	−0.0409 (9)
N1B	0.0166 (7)	0.0163 (6)	0.0215 (6)	0.0002 (5)	−0.0045 (5)	−0.0092 (5)
N2B	0.0168 (7)	0.0155 (6)	0.0179 (6)	−0.0016 (5)	−0.0018 (5)	−0.0074 (5)
N3B	0.0181 (7)	0.0175 (6)	0.0179 (6)	−0.0006 (5)	−0.0027 (5)	−0.0072 (5)
N4B	0.0183 (7)	0.0177 (6)	0.0205 (6)	−0.0011 (5)	−0.0024 (5)	−0.0080 (5)
N5B	0.0194 (7)	0.0267 (7)	0.0236 (7)	−0.0007 (6)	−0.0032 (5)	−0.0146 (6)
C1B	0.0248 (9)	0.0233 (8)	0.0197 (8)	−0.0062 (7)	−0.0030 (6)	−0.0054 (7)
C2B	0.0301 (10)	0.0198 (8)	0.0249 (8)	−0.0090 (7)	−0.0077 (7)	−0.0033 (7)
C3B	0.0256 (9)	0.0190 (8)	0.0252 (8)	−0.0015 (6)	−0.0104 (7)	−0.0084 (7)
C4B	0.0212 (8)	0.0213 (8)	0.0195 (7)	0.0000 (6)	−0.0047 (6)	−0.0073 (6)
C5B	0.0178 (8)	0.0177 (7)	0.0201 (7)	−0.0008 (6)	−0.0018 (6)	−0.0067 (6)
C6B	0.0172 (8)	0.0178 (7)	0.0197 (7)	−0.0008 (6)	−0.0026 (6)	−0.0079 (6)
C7B	0.0187 (8)	0.0229 (8)	0.0199 (7)	−0.0025 (6)	−0.0010 (6)	−0.0109 (6)
C8B	0.0135 (7)	0.0193 (7)	0.0197 (7)	0.0011 (6)	−0.0014 (5)	−0.0087 (6)
C9B	0.0178 (8)	0.0261 (8)	0.0200 (7)	0.0006 (6)	−0.0062 (6)	−0.0113 (7)
C10B	0.0319 (10)	0.0268 (9)	0.0360 (10)	0.0005 (8)	−0.0028 (8)	−0.0184 (8)
C11B	0.0349 (12)	0.0411 (12)	0.0560 (14)	0.0033 (9)	−0.0026 (10)	−0.0337 (11)
C12B	0.0234 (10)	0.0574 (14)	0.0424 (11)	−0.0027 (9)	0.0007 (8)	−0.0352 (11)
C13B	0.0202 (9)	0.0329 (10)	0.0279 (9)	−0.0026 (7)	−0.0010 (7)	−0.0169 (8)
C14B	0.0148 (7)	0.0152 (7)	0.0209 (7)	0.0004 (6)	−0.0008 (6)	−0.0082 (6)
C15B	0.0177 (8)	0.0170 (7)	0.0187 (7)	−0.0021 (6)	0.0002 (6)	−0.0072 (6)
C16B	0.0192 (8)	0.0132 (7)	0.0185 (7)	−0.0023 (6)	−0.0020 (6)	−0.0051 (6)
C17B	0.0213 (8)	0.0234 (8)	0.0200 (8)	−0.0001 (6)	−0.0030 (6)	−0.0105 (6)
C18B	0.0186 (8)	0.0252 (9)	0.0221 (8)	−0.0008 (6)	0.0006 (6)	−0.0105 (7)
C19B	0.0215 (8)	0.0147 (7)	0.0209 (7)	−0.0050 (6)	−0.0029 (6)	−0.0039 (6)
C20B	0.0291 (9)	0.0222 (8)	0.0205 (8)	0.0006 (7)	−0.0071 (7)	−0.0103 (7)
C21B	0.0267 (9)	0.0228 (8)	0.0194 (8)	0.0005 (7)	−0.0009 (6)	−0.0106 (7)
C22B	0.0196 (8)	0.0206 (8)	0.0244 (8)	−0.0048 (6)	−0.0042 (6)	−0.0065 (7)
C23B	0.0172 (8)	0.0201 (8)	0.0205 (7)	−0.0007 (6)	−0.0036 (6)	−0.0080 (6)
C24B	0.0262 (9)	0.0206 (8)	0.0294 (9)	−0.0028 (7)	−0.0059 (7)	−0.0095 (7)
C25B	0.0225 (9)	0.0279 (9)	0.0289 (9)	0.0011 (7)	−0.0076 (7)	−0.0110 (7)
C26B	0.0227 (9)	0.0223 (8)	0.0248 (8)	−0.0063 (7)	−0.0001 (6)	−0.0083 (7)

### Geometric parameters (Å, °)

**Table:**

Br1A—C3A	1.8975 (18)	Br1B—C3B	1.9007 (17)
S1A—C8A	1.6707 (17)	S1B—C8B	1.6740 (16)
O1A—C12A	1.425 (3)	O1B—C12B	1.420 (3)
O1A—C11A	1.429 (2)	O1B—C11B	1.423 (3)
N1A—C7A	1.275 (2)	N1B—C7B	1.278 (2)
N1A—N2A	1.389 (2)	N1B—N2B	1.3946 (18)
N2A—C14A	1.387 (2)	N2B—C14B	1.382 (2)
N2A—C8A	1.392 (2)	N2B—C8B	1.386 (2)
N3A—C8A	1.351 (2)	N3B—C8B	1.351 (2)
N3A—N4A	1.3850 (19)	N3B—N4B	1.3853 (18)
N3A—C9A	1.470 (2)	N3B—C9B	1.468 (2)
N4A—C14A	1.295 (2)	N4B—C14B	1.300 (2)
N5A—C9A	1.436 (2)	N5B—C9B	1.444 (2)
N5A—C13A	1.462 (2)	N5B—C13B	1.462 (2)
N5A—C10A	1.470 (2)	N5B—C10B	1.466 (2)
C1A—C2A	1.378 (3)	C1B—C2B	1.383 (2)
C1A—C6A	1.399 (2)	C1B—C6B	1.394 (2)
C1A—H1AA	0.93	C1B—H1BA	0.93
C2A—C3A	1.390 (3)	C2B—C3B	1.381 (2)
C2A—H2AA	0.93	C2B—H2BA	0.93
C3A—C4A	1.382 (2)	C3B—C4B	1.389 (2)
C4A—C5A	1.386 (3)	C4B—C5B	1.382 (2)
C4A—H4AA	0.93	C4B—H4BA	0.93
C5A—C6A	1.393 (2)	C5B—C6B	1.397 (2)
C5A—H5AA	0.93	C5B—H5BA	0.93
C6A—C7A	1.466 (2)	C6B—C7B	1.462 (2)
C7A—H7AA	0.93	C7B—H7BA	0.93
C9A—H9AA	0.97	C9B—H9BA	0.97
C9A—H9AB	0.97	C9B—H9BB	0.97
C10A—C11A	1.507 (3)	C10B—C11B	1.509 (3)
C10A—H10A	0.97	C10B—H10C	0.97
C10A—H10B	0.97	C10B—H10D	0.97
C11A—H11A	0.97	C11B—H11C	0.97
C11A—H11B	0.97	C11B—H11D	0.97
C12A—C13A	1.512 (3)	C12B—C13B	1.512 (3)
C12A—H12A	0.97	C12B—H12C	0.97
C12A—H12B	0.97	C12B—H12D	0.97
C13A—H13A	0.97	C13B—H13C	0.97
C13A—H13B	0.97	C13B—H13D	0.97
C14A—C15A	1.495 (2)	C14B—C15B	1.496 (2)
C15A—C16A	1.523 (2)	C15B—C16B	1.514 (2)
C15A—C26A	1.531 (2)	C15B—C26B	1.533 (2)
C15A—H15A	0.98	C15B—H15B	0.98
C16A—C17A	1.391 (2)	C16B—C21B	1.391 (2)
C16A—C21A	1.394 (2)	C16B—C17B	1.392 (2)
C17A—C18A	1.390 (3)	C17B—C18B	1.388 (2)
C17A—H17A	0.93	C17B—H17B	0.93
C18A—C19A	1.385 (3)	C18B—C19B	1.390 (2)
C18A—H18A	0.93	C18B—H18B	0.93
C19A—C20A	1.396 (3)	C19B—C20B	1.394 (2)
C19A—C22A	1.509 (3)	C19B—C22B	1.506 (2)
C20A—C21A	1.390 (3)	C20B—C21B	1.388 (3)
C20A—H20A	0.93	C20B—H20B	0.93
C21A—H21A	0.93	C21B—H21B	0.93
C22A—C23A	1.525 (3)	C22B—C23B	1.538 (2)
C22A—H22A	0.97	C22B—H22C	0.97
C22A—H22B	0.97	C22B—H22D	0.97
C23A—C25A	1.503 (4)	C23B—C24B	1.522 (2)
C23A—C24A	1.532 (3)	C23B—C25B	1.528 (2)
C23A—H23A	0.98	C23B—H23B	0.98
C24A—H24A	0.96	C24B—H24D	0.96
C24A—H24B	0.96	C24B—H24E	0.96
C24A—H24C	0.96	C24B—H24F	0.96
C25A—H25A	0.96	C25B—H25D	0.96
C25A—H25B	0.96	C25B—H25E	0.96
C25A—H25C	0.96	C25B—H25F	0.96
C26A—H26A	0.96	C26B—H26D	0.96
C26A—H26B	0.96	C26B—H26E	0.96
C26A—H26C	0.96	C26B—H26F	0.96
			
C12A—O1A—C11A	109.42 (15)	C12B—O1B—C11B	109.53 (16)
C7A—N1A—N2A	118.96 (15)	C7B—N1B—N2B	116.17 (13)
C14A—N2A—N1A	118.48 (14)	C14B—N2B—C8B	108.67 (13)
C14A—N2A—C8A	108.24 (14)	C14B—N2B—N1B	119.85 (13)
N1A—N2A—C8A	132.91 (14)	C8B—N2B—N1B	131.46 (13)
C8A—N3A—N4A	113.15 (14)	C8B—N3B—N4B	113.52 (13)
C8A—N3A—C9A	127.36 (14)	C8B—N3B—C9B	125.58 (14)
N4A—N3A—C9A	118.31 (14)	N4B—N3B—C9B	120.31 (13)
C14A—N4A—N3A	105.00 (13)	C14B—N4B—N3B	104.42 (13)
C9A—N5A—C13A	113.45 (14)	C9B—N5B—C13B	113.07 (14)
C9A—N5A—C10A	114.09 (14)	C9B—N5B—C10B	113.21 (14)
C13A—N5A—C10A	111.22 (14)	C13B—N5B—C10B	110.14 (15)
C2A—C1A—C6A	120.34 (16)	C2B—C1B—C6B	120.78 (16)
C2A—C1A—H1AA	119.8	C2B—C1B—H1BA	119.6
C6A—C1A—H1AA	119.8	C6B—C1B—H1BA	119.6
C1A—C2A—C3A	119.29 (17)	C3B—C2B—C1B	118.68 (16)
C1A—C2A—H2AA	120.4	C3B—C2B—H2BA	120.7
C3A—C2A—H2AA	120.4	C1B—C2B—H2BA	120.7
C4A—C3A—C2A	121.59 (17)	C2B—C3B—C4B	121.87 (16)
C4A—C3A—Br1A	118.94 (13)	C2B—C3B—Br1B	118.95 (13)
C2A—C3A—Br1A	119.46 (13)	C4B—C3B—Br1B	119.18 (13)
C3A—C4A—C5A	118.64 (16)	C5B—C4B—C3B	118.93 (15)
C3A—C4A—H4AA	120.7	C5B—C4B—H4BA	120.5
C5A—C4A—H4AA	120.7	C3B—C4B—H4BA	120.5
C4A—C5A—C6A	120.98 (16)	C4B—C5B—C6B	120.32 (15)
C4A—C5A—H5AA	119.5	C4B—C5B—H5BA	119.8
C6A—C5A—H5AA	119.5	C6B—C5B—H5BA	119.8
C5A—C6A—C1A	119.16 (16)	C1B—C6B—C5B	119.38 (15)
C5A—C6A—C7A	118.04 (15)	C1B—C6B—C7B	118.38 (15)
C1A—C6A—C7A	122.80 (16)	C5B—C6B—C7B	122.24 (15)
N1A—C7A—C6A	119.92 (16)	N1B—C7B—C6B	120.00 (15)
N1A—C7A—H7AA	120.0	N1B—C7B—H7BA	120.0
C6A—C7A—H7AA	120.0	C6B—C7B—H7BA	120.0
N3A—C8A—N2A	102.82 (14)	N3B—C8B—N2B	102.52 (13)
N3A—C8A—S1A	126.71 (13)	N3B—C8B—S1B	127.83 (12)
N2A—C8A—S1A	130.43 (13)	N2B—C8B—S1B	129.48 (12)
N5A—C9A—N3A	116.28 (14)	N5B—C9B—N3B	114.70 (13)
N5A—C9A—H9AA	108.2	N5B—C9B—H9BA	108.6
N3A—C9A—H9AA	108.2	N3B—C9B—H9BA	108.6
N5A—C9A—H9AB	108.2	N5B—C9B—H9BB	108.6
N3A—C9A—H9AB	108.2	N3B—C9B—H9BB	108.6
H9AA—C9A—H9AB	107.4	H9BA—C9B—H9BB	107.6
N5A—C10A—C11A	110.09 (15)	N5B—C10B—C11B	109.00 (17)
N5A—C10A—H10A	109.6	N5B—C10B—H10C	109.9
C11A—C10A—H10A	109.6	C11B—C10B—H10C	109.9
N5A—C10A—H10B	109.6	N5B—C10B—H10D	109.9
C11A—C10A—H10B	109.6	C11B—C10B—H10D	109.9
H10A—C10A—H10B	108.2	H10C—C10B—H10D	108.3
O1A—C11A—C10A	110.79 (15)	O1B—C11B—C10B	111.30 (17)
O1A—C11A—H11A	109.5	O1B—C11B—H11C	109.4
C10A—C11A—H11A	109.5	C10B—C11B—H11C	109.4
O1A—C11A—H11B	109.5	O1B—C11B—H11D	109.4
C10A—C11A—H11B	109.5	C10B—C11B—H11D	109.4
H11A—C11A—H11B	108.1	H11C—C11B—H11D	108.0
O1A—C12A—C13A	110.22 (16)	O1B—C12B—C13B	111.70 (17)
O1A—C12A—H12A	109.6	O1B—C12B—H12C	109.3
C13A—C12A—H12A	109.6	C13B—C12B—H12C	109.3
O1A—C12A—H12B	109.6	O1B—C12B—H12D	109.3
C13A—C12A—H12B	109.6	C13B—C12B—H12D	109.3
H12A—C12A—H12B	108.1	H12C—C12B—H12D	107.9
N5A—C13A—C12A	109.19 (15)	N5B—C13B—C12B	110.45 (15)
N5A—C13A—H13A	109.8	N5B—C13B—H13C	109.6
C12A—C13A—H13A	109.8	C12B—C13B—H13C	109.6
N5A—C13A—H13B	109.8	N5B—C13B—H13D	109.6
C12A—C13A—H13B	109.8	C12B—C13B—H13D	109.6
H13A—C13A—H13B	108.3	H13C—C13B—H13D	108.1
N4A—C14A—N2A	110.74 (15)	N4B—C14B—N2B	110.69 (14)
N4A—C14A—C15A	126.11 (15)	N4B—C14B—C15B	126.33 (14)
N2A—C14A—C15A	123.15 (15)	N2B—C14B—C15B	122.97 (14)
C14A—C15A—C16A	111.78 (14)	C14B—C15B—C16B	111.17 (13)
C14A—C15A—C26A	110.46 (15)	C14B—C15B—C26B	110.32 (14)
C16A—C15A—C26A	111.11 (14)	C16B—C15B—C26B	111.12 (13)
C14A—C15A—H15A	107.8	C14B—C15B—H15B	108.0
C16A—C15A—H15A	107.8	C16B—C15B—H15B	108.0
C26A—C15A—H15A	107.8	C26B—C15B—H15B	108.0
C17A—C16A—C21A	118.32 (16)	C21B—C16B—C17B	118.16 (16)
C17A—C16A—C15A	120.15 (16)	C21B—C16B—C15B	120.75 (15)
C21A—C16A—C15A	121.51 (16)	C17B—C16B—C15B	121.05 (14)
C18A—C17A—C16A	120.44 (17)	C18B—C17B—C16B	120.85 (16)
C18A—C17A—H17A	119.8	C18B—C17B—H17B	119.6
C16A—C17A—H17A	119.8	C16B—C17B—H17B	119.6
C19A—C18A—C17A	121.61 (17)	C17B—C18B—C19B	121.25 (16)
C19A—C18A—H18A	119.2	C17B—C18B—H18B	119.4
C17A—C18A—H18A	119.2	C19B—C18B—H18B	119.4
C18A—C19A—C20A	117.92 (17)	C18B—C19B—C20B	117.74 (16)
C18A—C19A—C22A	120.89 (17)	C18B—C19B—C22B	121.30 (16)
C20A—C19A—C22A	121.20 (17)	C20B—C19B—C22B	120.97 (15)
C21A—C20A—C19A	120.80 (16)	C21B—C20B—C19B	121.21 (16)
C21A—C20A—H20A	119.6	C21B—C20B—H20B	119.4
C19A—C20A—H20A	119.6	C19B—C20B—H20B	119.4
C20A—C21A—C16A	120.90 (16)	C20B—C21B—C16B	120.78 (16)
C20A—C21A—H21A	119.5	C20B—C21B—H21B	119.6
C16A—C21A—H21A	119.5	C16B—C21B—H21B	119.6
C19A—C22A—C23A	114.42 (17)	C19B—C22B—C23B	114.05 (13)
C19A—C22A—H22A	108.7	C19B—C22B—H22C	108.7
C23A—C22A—H22A	108.7	C23B—C22B—H22C	108.7
C19A—C22A—H22B	108.7	C19B—C22B—H22D	108.7
C23A—C22A—H22B	108.7	C23B—C22B—H22D	108.7
H22A—C22A—H22B	107.6	H22C—C22B—H22D	107.6
C25A—C23A—C22A	112.0 (2)	C24B—C23B—C25B	111.57 (14)
C25A—C23A—C24A	111.9 (2)	C24B—C23B—C22B	110.81 (14)
C22A—C23A—C24A	109.77 (19)	C25B—C23B—C22B	109.58 (14)
C25A—C23A—H23A	107.7	C24B—C23B—H23B	108.3
C22A—C23A—H23A	107.7	C25B—C23B—H23B	108.3
C24A—C23A—H23A	107.7	C22B—C23B—H23B	108.3
C23A—C24A—H24A	109.5	C23B—C24B—H24D	109.5
C23A—C24A—H24B	109.5	C23B—C24B—H24E	109.5
H24A—C24A—H24B	109.5	H24D—C24B—H24E	109.5
C23A—C24A—H24C	109.5	C23B—C24B—H24F	109.5
H24A—C24A—H24C	109.5	H24D—C24B—H24F	109.5
H24B—C24A—H24C	109.5	H24E—C24B—H24F	109.5
C23A—C25A—H25A	109.5	C23B—C25B—H25D	109.5
C23A—C25A—H25B	109.5	C23B—C25B—H25E	109.5
H25A—C25A—H25B	109.5	H25D—C25B—H25E	109.5
C23A—C25A—H25C	109.5	C23B—C25B—H25F	109.5
H25A—C25A—H25C	109.5	H25D—C25B—H25F	109.5
H25B—C25A—H25C	109.5	H25E—C25B—H25F	109.5
C15A—C26A—H26A	109.5	C15B—C26B—H26D	109.5
C15A—C26A—H26B	109.5	C15B—C26B—H26E	109.5
H26A—C26A—H26B	109.5	H26D—C26B—H26E	109.5
C15A—C26A—H26C	109.5	C15B—C26B—H26F	109.5
H26A—C26A—H26C	109.5	H26D—C26B—H26F	109.5
H26B—C26A—H26C	109.5	H26E—C26B—H26F	109.5

### Hydrogen-bond geometry (Å, °)

**Table:**

*D*—H···*A*	*D*—H	H···*A*	*D*···*A*	*D*—H···*A*
C7A—H7AA···S1A	0.93	2.46	3.195 (2)	137
C9A—H9AB···S1A	0.97	2.86	3.252 (2)	105
C4B—H4BA···N5A^i^	0.93	2.56	3.384 (2)	147
C10A—H10A···Br1A^ii^	0.97	2.86	3.770 (2)	158
C7B—H7BA···S1B	0.93	2.56	3.190 (2)	125
C9B—H9BB···S1B	0.97	2.85	3.254 (2)	106
C15A—H15A···O1A^iii^	0.98	2.29	3.244 (2)	165
C4A—H4AA···Cg1^ii^	0.93	2.53	3.401 (2)	156

Symmetry codes: (i) *x*, *y*, *z*+1; (ii) −*x*+2, −*y*, −*z*; (iii) *x*+1, *y*, *z*.
